# Association of COVID-19 With Emergence of Comorbidities: A Hospital-Based Cohort Study

**DOI:** 10.7759/cureus.66147

**Published:** 2024-08-04

**Authors:** Khushi Nayyar, Tanvir K Sidhu, Avneet K Garg

**Affiliations:** 1 Internal Medicine, Adesh Institute of Medical Sciences and Research, Bathinda, IND; 2 Community Medicine, Adesh Institute of Medical Sciences and Research, Bathinda, IND; 3 Pulmonary Medicine, Adesh Institute of Medical Sciences and Research, Bathinda, IND

**Keywords:** complications, post-exposure, covid-19, pandemic, comorbidities

## Abstract

Background: In 2019, the emergence of SARS-CoV-2 marked the beginning of the COVID-19 global pandemic, which reached its peak in 2020. Initially designated as a novel coronavirus, SARS-CoV-2 emerged as a respiratory illness and later began causing multi-organ complications in recovered patients.

Methods: This article presents a hospital-based retrospective cohort study conducted via telephone interviews with patients in a tertiary hospital. After obtaining verbal consent from the subjects, the study utilized a semi-structured questionnaire to gather data.

Results: In the 54-person cohort group, 64.8% were males and 35.1% were females. The mean duration of the male patients’ hospital stays was greater than that of the female patients. However, the mean lag time between the onset of comorbidities and recovery from COVID-19 was shorter in females than in males. Upon further analysis, it was revealed that female patients are more susceptible to the development of multiple comorbidities at once, occurring in 37.5% of the female patients in this study. Diabetes mellitus alone had the highest incidence rate (12.9%), followed by ST-elevation myocardial Infarctions (7.4%) and thrombocytopenia (5.5%). Of the cohort group, 51.8% developed comorbidities after exposure to COVID-19, while about 14.8% of the control group developed comorbidities from March 2020 onwards, i.e. from the commencement of the COVID-19 global pandemic. The relative risk assessed for this study is 3.5. The study’s attributable risk is 71.42%.

Conclusion: The incidence of comorbidities in the cohort group was greater than that in the control group, demonstrating COVID-19 as a risk factor for post-exposure comorbidities. It is clear that there is a direct association between COVID-19 and the development of comorbidities, which is inferred with a relative risk of 3.5.

## Introduction

SARS-CoV-2 emerged in late 2019 and was declared a global pandemic in March 2020 [1]. SARS-CoV-2 emerged as a respiratory infection of viral etiology, but with time, it became evident that the virus triggered significant changes in the body during the convalescent and recovery stages of the disease [2]. The rise of comorbidities in COVID-recovered patients set off a wave of new research.

Much research has been presented since the pandemic’s inception. Various complications have arisen in individuals who were infected by or have recovered from COVID-19, some leading to chronic disability or death [3], affecting quality of life, or causing autonomic dysfunction [4]. Several investigational studies have drawn inferences regarding post-COVID-19 complications, many pointing to the associative risk factors of age and chronic metabolic conditions. Such risk factors can aggravate the potential for complications that can lead to the formation of a vicious cycle of comorbidities [5]. Immuno-compromised populations are more susceptible to complications from SARS-CoV-2 [6].

This study aims to examine the incidence and changing patterns of comorbidities in COVID-recovered patients with respect to age and sex. Data analysis conducted in both the cohort and the control group will be presented to make a case for the plausible association of comorbidities in COVID-19.

The objective of this study is to examine the incidence of comorbidities in patients who have recovered from COVID-19 and to determine whether comorbidities are associated with COVID-19 infection.

The null hypothesis of this research is that there is no increase in comorbidities in patients who have recovered from COVID-19 compared with the general population.

The alternative hypothesis of this research is there is a positive association of comorbidities in patients who have recovered from COVID-19.

## Materials and methods

Study design

This is a retrospective observational cohort study conducted in a tertiary care hospital (Adesh Institute of Medical Sciences and Research, Bathinda) in the state of Punjab, India. The study examines a period of time spanning from participants’ first infection with COVID-19 to their outcome from the disease and analyzes any comorbidities they developed. The risk factor assessed in this study was infection with COVID-19 disease.

Participants

This study had a sample size of 108 individuals divided into a cohort group and a control group by a 1:1 ratio. The cohort group included 54 patients admitted to a tertiary care hospital in Punjab, India with a positive COVID test between March 2020 and February 2022. Of the cohort group, 64.8% were men and 35.1% were women. The control group was a sample population of 54 age- and sex-matched family members and relatives of the cohort group who were negative for COVID-19. All information was recorded using a case report form and a questionnaire including relevant parameters.

Methodology

A semi-structured questionnaire was prepared for data collection by telephone interview. After explaining the purpose of the study to all study participants in their vernacular language and maintaining their proper anonymity, the verbal consent of all study participants was obtained for the interview. It was made clear that study participants reserved the right to drop out of the study at any time without explanation.

The cohort group was questioned about any sequelae they observed following infection with COVID-19. To collect information about the control group, the cohort group was asked about their family members & relatives of the same demographic (i.e., age and sex) who were COVID-negative and whether they had suffered from any comorbidities from March 2020 onwards (i.e., since the commencement of the COVID-19 global pandemic). Cohort group members were contacted to collect the relevant information.

The inclusion criteria for the cohort group were as follows: (1) a positive COVID-19 RT-PCR test documented by hospital lab reports and (2) admittance to the study hospital as an inpatient with complete data records available. The inclusion criteria for the control group were as follows: (1) relatives and family members of the cohort group who were COVID-negative (e.g., via PCR test/ Rapid Antigen test) and (2) relatives and family members who were a demographic match to the cohort group by age and sex. The exclusion criteria for the cohort group were as follows: (1) the presence of comorbidities prior to COVID-19 infection, (2) individuals unwilling to share information or from whom consent had not been obtained, and (3) those who could not be contacted by telephone after three repeated attempts. The exclusion criteria for the control group were as follows: (1) the presence of any comorbidities prior to March 2020 and (2) those who were not willing to share information or from whom consent had not been obtained. Microsoft Excel and openepi. com were used for data analysis. Ethical approval for this study was obtained from the Ethical Committee of Adesh Institute of Medical Sciences and Research (Reference no: AU/EC_BHR/2K23/349).

## Results

The mean age of the cohort group in this study was 55.5 ± 14.95 y. The mean age among men was 54.5 ± 13.4 y, and the mean age among women was 55.5 ± 16.7 y. The mean duration of hospital stay for the cohort group was 7 ± 4.63 days. By gender, the mean duration of hospital stay was 8.1 ± 4.6 days among men and 6.5 ± 3.5 days among women. Thus, men showed an increased duration of the disease state. The development of comorbidities was greatest for those with a hospital stay of 6-10 days, followed by those with a hospital stay of 1-5 days (Table 1).

**Table 1:** Comparison between comorbidities by gender and duration of hospital stay in the cohort group

Severity of illness based on the duration of hospital stay (in days)	Comorbidities among men	Comorbidities among women	Total comorbidities
1–5	6	1	7
6–10	9	5	14
11–15	3	2	5
16–20	1	–	1
21–25	1	–	1

The mean recovery time in days was 18 ± 25.02. The mean recovery time was 21.1 ± 30.0 days in men and 12.1 ± 8.0 days in women. However, the mean time lag between COVID-19 recovery and the onset of comorbidities was 42 ± 80.78 days and was shorter in women (59.5 ± 37.5 days) compared with men (88.5 ± 110.2 days).

Of the cohort group, 51.8% developed comorbidities following infection with COVID-19, while only 14.8% of the control group developed comorbidities from March 2020 onwards (Table 2).

**Table 2:** Incidence of comorbidities in each group

	Comorbidities present	Comorbidities absent	Total
Cohort group	28	26	54
Control group	8	46	54
Total	36	72	108

Relative risk was calculated at 3.5 with a CI of 95%, meaning that the risk of developing comorbidities was 3.5× greater in the cohort group than in the control group. Attributable risk, the proportion of disease that is attributable to infection, was calculated at 71.42%, demonstrating the direct association of COVID-19 infection with the development of comorbidities.

An analysis of the development of multiple comorbidities after COVID-19 infection revealed that women were more prone to developing multiple comorbidities simultaneously, as 37.5% of women developed comorbidities, while only 5% of men developed multiple comorbidities. Diabetes mellitus was the comorbidity with the highest incidence rate independently, at 12.9%, followed by ST-elevation myocardial infarction (STEMI) at 7.4% and thrombocytopenia at 5.5% (Table 3).

**Table 3:** Incidence of comorbidities in both groups STEMI: ST elevation myocardial infarction; NSTEMI: non-ST elevation myocardial infarction; TIA: transient ischemic attack. *Indicates multiple comorbidities.

Comorbidities	N=54[cohort group]	% age in the cohort group	N=54[control group]	%age in the control group
Diabetes mellitus	7	12.9	4	7.4
STEMI	4	7.4	1	1.8
NSTEMI	1	1.8	_	_
Ischemic stroke	2	3.7	_	_
Hypotension	1	1.8	_	_
Thrombocytopenia	3	5.5	_	_
Hypertension	2	3.7	_	_
Pulmonary tuberculosis	1	1.8	_	_
Asthma	1	1.8	2	3.7
Pneumonia	1	1.8	_	_
TIA	1	1.8	_	_
Diabetes mellitus and STEMI*	2	3.7	_	_
NSTEMI and hypertension*	1	1.8	_	_
Asthma and thrombocytopenia*	1	1.8	_	_

## Discussion

The total prevalence of comorbidities following COVID-19 infection among participants in the cohort group of the current study was 51.8%, compared with 14.8% in the control group and 42% in a different study [7]. This may be explained by the fact that the reference research was conducted in the year 2020, while the current study is a retrospective cohort completed in the year 2023, so the number of comorbidities has increased with the years.

Within the cohort group, 64.8% were men and 35.1% were women. Thus, men were already affected by COVID-19 at a greater rate than women. This may be due to their increased vulnerability in developing countries, wherein men generally face greater exposure to the external environment than women. It may also be that more men are admitted to the hospital setting in general. The results also showed an increased duration of the disease state in men, with a mean duration of hospital stay of 8.1 ± 4.6 days, compared with 6.5 ± 3.5 days among women, and a better mean recovery time of 12.1 ± 8.0 days among women, versus 21.1 ± 30.0 days among men. This may be related to the severity of the disease in men compared with women, as well as to the fact that longer hospital stays are associated with better treatment and supervision by healthcare staff. It may also be a consequence of women’s earlier voluntary discharge from the hospital setting due to social and domestic factors.

Upon further analysis of age relation, it can be concluded that the incidence of comorbidities after COVID-19 infection increases with advancing age. The incidence of comorbidities was 7.14% in the <40 y age group, 35.7% in the 40-60 y age group, and 57.1% in the >60 y age group. There is an increased prevalence of independent and combined comorbidities in older age groups. The incidence of multiple comorbidities was 18.7% for those >60 y of age, versus 8.3% for those <60 y of age. This is suggestive of poor immunity and increased susceptibility to disease among the elderly.

Diabetes mellitus had the highest incidence rate (12.9%) of all morbidities, followed by STEMI (7.4%) and thrombocytopenia (5.5%) (Table 3). This corroborates the results of another study, which discusses the increased risk of thrombotic activity in patients with severe symptoms of COVID-19 [8,9]. Additionally, the risk of developing venous thromboembolism is greater in ICU patients, as per another study [10]. It has been stated that the incidence of thrombocytopenia increases the risk of immune thrombocytopenic purpura in patients with COVID-19 [11]. Regarding other comorbidities in patients with COVID-19, other studies report the incidence of diabetes mellitus at 14.4% [12] and 36.7% [13]; of ischemic stroke at 2% [14], 4.6% [15], and 5% [16]; of hypotension at 37.5% [17]; of pneumonia at 20% [18]; of asthma at 4.3% [19]; and of hypertension at 32% [7]; of STEMI at 5% [20]; and 43.3% [13].

The increased incidence of diabetes mellitus observed may be due to the continued use of steroids in the treatment of COVID-19. Most of the complications of diabetes mellitus are due to indiscriminate use of glucocorticoids and the dysfunction of beta cells due to COVID-19 leading to diabetes [21].

## Conclusions

The incidence of comorbidities in the cohort group of the current study was significantly higher than in the control group, demonstrating that there is direct association between COVID-19 infection and the development of comorbidities. The prevalence and severity of comorbidities increased according to age and gender and was higher among the elderly and women. Overall, diabetes mellitus had the highest independent incidence rate, followed by STEMI and thrombocytopenia. As many comorbidities have long latency periods, future research should focus on tracking the pattern of developing diseases more precisely over the years and in other states in India.

## Appendices

**Table 4:** Demographic details of both groups

Mean age (y)			55.5 ± 14.95	54.7 ± 15.04
Sex	M		35	35
	F		19	19
Location	Rural		26	15
	Urban		28	39
Reported comorbidities			28	8
Management of comorbidities		Medical	20	7
		Surgical	6	1
		Both	2	0
No. of patients on regular medications			20	8

**Table 5:** Questionnaire data DM: Diabetes mellitus; STEMI: ST elevation myocardial infarction; TIA: transient ischemic attack

Serial number	Questions	Response
1	Did you have symptoms of COVID-19 or asymptomatic?	Yes/no (if yes, tell the symptoms)
2	How long did it take to recover from COVID-19?	Give in days/weeks
3	Any complications developed after COVID-19?	ex. DM, STEMI, TIA, etc.
4	What was/were management of the complication(s)?	Medical/surgical intervention
5	After how long post COVID-19 recovery did complications develop?	Give in days/weeks
6	What is the current status of the complication(s)?	If on regular medication or not
7	(For Control) Name of any family member / relative of same age & sex but not exposed to COVID-19 infection	Provide relevant with name
8	Whether the control member/control relative has some non-communicable disease or not?	Yes/no (if yes, provide names)

**Table 6:** Case record form data

Serial number	Case record form data	Patient’s information
1	Name	
2	Address	
3	Age	
4	Sex	
5	Mobile number	
6	Date of visit to hospital	
7	Report information: (select the investigations done to declare COVID-19 positive)	RTPCR/Rapid antigen test (select one)
8	Number of days of stay in the hospital	
9	Severity of disease	Whether ventilated or not?
